# Smallpox in England and Wales

**Published:** 1924-10

**Authors:** 


### SMALLPOX IN ENGLAND AND WALES.

On September 18 the following notification was issued by the Ministry of Health :—At the present time smallpox is prevalent in the County Borough of Middlesbrough, the Borough of Chesterfield and the Urban District of Hucknall, in all of which districts the disease has continued to be present since the autumn of 1923. Other sanitary districts now mainly affected are the County Borough of Derby, the Rural District of Chesterfield, the Borough of Mansfield and the Urban Districts of Mansfield Woodhouse, Ashington, Melton Mowbray and Gainsborough. Within the last four week sporadic cases have occurred in 21 other sanitary districts, including the Metropolitan Borough of St. Pancras, the County Boroughs of Barnsley, Leeds, Leicester, Sheffield and Oxford. On three separate occasions this year a single case of the disease has been notified from different Metropolitan Boroughs.

In 1924 up to the end of the second week in September, smallpox has appeared in 91 sanitary districts in England and Wales, including four port sanitary districts. The total number of cases is approximately 2,750 as compared with 1,840 in the corresponding period of 1923. The county most heavily invaded is Derby with a total of approximately 1,080 cases in sixteen sanitary districts ; the next in point of number is the contiguous county of Nottingham with 434 cases in ten sanitary districts ; 386 cases have occurred in Yorkshire (mainly in Middlesbrough) and 306 in Northumberland (mainly in Ashington). In the Willesden Urban District in June-July there were ten cases, with three deaths.

